# Poly-guanidine shows high cytotoxicity in glioma cell cultures and glioma stem cells

**DOI:** 10.1007/s10637-022-01233-7

**Published:** 2022-03-21

**Authors:** Marcela Márquez, Karl Holmberg Olausson, Ayodele Alaiya, Sten Nilsson, Lennart Meurling, Anders R. Holmberg

**Affiliations:** 1 Department of Oncology-Pathology, Karolinska Institute, Stockholm, Sweden; 2 Universidad Autónoma de Nuevo León, Centro de Investigación Y Desarrollo en Ciencias de La Salud, Monterrey, Nuevo Leon Mexico; 3 Proteomic Unit, Stem Cell and Tissue Re-Engineering Program, King Faisal Specialist Hospital and Research Center, Riyadh, Saudi Arabia

**Keywords:** Poly-guanidine, Glioma cells, Patient-derived glioma cell lines (PDGCLs), Sialic acid, Cell-killing efficacy, New spheroid fluorometric cytotoxicity assay

## Abstract

Glioblastoma multiforme (GBM) is a malignant CNS tumor with a poor prognosis. GBM shows aberrant glycosylation with hypersialylation. This property is a potential target for therapy. This study investigates the growth inhibitory efficacy of poly-guanidine (GuaDex), with an affinity for sialic acid (Sia). Glioma cell cultures and patient-derived glioma cell lines (PDGCLs) expressing Prominin-1 (CD133) were used. Human fibroblasts and astrocyte-derived cells were used as controls. Temozolomide (standard GBM drug, TMZ) and DMSO were used as a comparison. GuaDex at 1–10 µM concentrations, were incubated for 3.5–72 h and with PDGCLs cells for 6–24 h. The cytotoxicity was estimated with a fluorometric cytotoxicity assay (FMCA). Fluorescence-labelled GuaDex was used to study the cell interactions. Sia expression was confirmed with a fluorescence labelled Sia binding lectin. Expression of glial fibrillary acidic protein was determined. GuaDex induction of growth inhibition was fast, showing after less than 5 min incubation while the control cells were not affected even after 50 min incubation. The growth inhibitory effect on PDGCLs spheroids was persistent still showing after 4 weeks post-treatment. The growth inhibition of GuaDex was induced at low µM concentrations while TMZ induced only a slight inhibition at mM concentrations. GuaDex efficacy appears significant and warrants further studies.

## Introduction

Glioblastoma multiforme (GBM) is a highly malignant central nervous system (CNS) tumor and remains incurable. The prognosis is bleak with less than 5% of diagnosed patients alive after 5 years. Median survival time post diagnosis is approximately 15 months. The presumed cellular origin is astrocytes and GBM is the most aggressive of astrocytic tumors. The etiology of glioma remains largely unknown. The vast majority of GBM is located in the supratentorial cerebral hemispheres and only a few percent in cerebellum, brainstem and spinal cord. Imaging techniques are used for diagnosis of gliomas, primarily magnetic resonance scanning (MR), also with gadolinium enhancement. Usual finding is a unifocal lesion with central necrosis surrounded by edema [1].

The principal treatment of GBM is surgery followed by radiation and chemotherapy. However, all treatment is restricted by three principal inherent factors, namely the tumors delicate location i.e., risking damage to healthy brain tissue resulting in devastating side effects, the blood brain barrier (BBB) and last, the properties of the tumor itself i.e., infiltrative growth and a general treatment resistance. Surgery will, importantly, temporarily alleviate symptoms (e.g., mass effects, neurological symptoms, seizures etc.) and like this improve quality of life of the patient. However, the infiltrative growth of GBM, leaving no clear border between tumor and normal brain tissue, precludes radical/curative surgery. Consequently, and despite added chemotherapy and radiation therapy, occurrence happens in most cases in and around the margin of the resected lesion [2]. Likewise, the delicacy of the surrounding healthy brain tissue and the tumors infiltrative growth, limits the effectiveness of radiotherapy to avoid severe side effects (radiation necrosis, nerve cell damage etc.). The BBB carefully regulates entrance to the parenchymal area of the CNS through its special semi permeable capillaries discriminating between molecular weight and chemical properties etc. [3]. Thus, BBB limits which drugs that may be used for systemic therapy. Nitrosourea compounds have been used to treat GBM however with transient and modest efficacy and with significant side effects [4]. Temozolomide (TMZ) is a triazene and is rapidly converted to an active form at physiologic pH to mono-methyl-triazene -imidazole- carboxamide (MTIC, a DNA methylator). TMZ is at the present the standard chemotherapeutic drug for systemic treatment of GBM [5]. The efficacy is modest and with several serious side effects. In addition, GBM appears in itself very resistant to any treatment apparent also when investigating efficacy of drugs in vitro and under ideal conditions. This was obvious also in this study.

Hence, the factors affecting treatment of GBM are unfavourable and new approaches are highly needed to improve the prognosis of this patient category.

Aberrant glycosylation resulting in hyper-sialylation is a general feature of malignant cells that differentiate them from normal cells. This feature may offer a target for specific treatment. Hyper-sialylation is advantageous to the tumor cells facilitating invasion/infiltration, evasion of the immune system and confers resistance to chemo/radiation therapy [6, 7].

One significant consequence of the hyper-sialylation is a strong anionic electrostatic charge, which may serve as a primary, and tumor selective target [8]. Cationic cytotoxic compounds such as poly-guanidine (GuaDex, by definition a polyamine) can be electrostatically attracted to the anionic tumor and be internalized via the polyamine uptake system and when intracellular, induce significant toxicity (9). The polyamine hunger expressed by malignant cells reflects their elevated metabolic needs [10, 11].

This in vitro study investigates the efficacy of GuaDex on GBM cell cultures and the efficacy is compared to the standard chemotherapeutic drug for treatment of GBM.

## Materials and methods

### Serum based cell culture of glioma cell lines

Glioma cell lines U373MG, U251MG, U1242MG, U343MG and U343MGa Cl2.6, (Table 1) were from the Prof. Monica Nister laboratory, CCK, Karolinska Institutet. The cells were cultured in Iscoves Modified Dulbeccos medium (IMDM) containing 10% fetal bovine serum, antibiotics (100 µg of penicillin and 50 µg of streptomycin sulphate/mL), and 2 mM glutamine at 37 °C, 5% CO.

**Table 1:** The human glioma cell lines and patient-derived glioma cell lines used in the experimental study

**Cell Lines**	**Characteristics**	**References**
U373 MG	GFAP positive	Westermark et al. [36] Westermark [37] Bongcam-R et al. [38]
U251MG	Astrocytoma, EGFR, GFAP positive	Bongcam-R et al. [38] Bigner et al. [39]
U1242MG	GFAP negative	Bongcam-R et al. [38] Nister et al. [40]
U343MG	GFAP negative	Bongcam-R et al. [38] Westermark et al. [36] Westermark [37] Nister et al. [41]
U343MGa CL2.6	Clonal derivative (CL2) of U343	Nister et al. [41]
	Produces a PDGF-like growth factor GFAP positive	Bongcam-R et al. [38]
**PDGCLs**		
BT112	High-grade, wild- type p53, Mutated or amplified EGFR	Mehta et al. [42]
	High Prominin-1 (CD133)	Holmberg O et al. [43]
BT179	Heterogenous phenotype, Mutated p53	Stevens et al. [44]
	Low Prominin-1 (CD133)	Holmberg O et al. [43]

*GFAP* glial fibrillary acidic protein, *PDGCLs* patient derived glioma cell lines, *PDGF* platelet derived growth factor, *EGFR* epidermal growth factor receptor

### Glioma patient-derived cell lines (PDGCLs)

BT112 and BT179 PDGCLs expressing Prominin-1 (CD133) (positive marker for stem cells) (Table 1) were a kind gift from Dr. Keith L. Ligon (Dana-Farber Cancer Institute, Harvard Medical School, Boston, MA, USA). Briefly, 500–1000 k PDGCLs cells were growth using Corning^®^ Ultra-Low attachment cell culture flasks 75 cm^2^ in Stem cell Technologies NeuroCult^®^ NS-A Proliferation Kit (Human) supplanted with 2 µg/mL Heparin, human rh EGF 20 ng/mL, human rh bFGF 20 ng/mL and antibiotics (100 µg penicillin and 50 µg streptomycin sulphate/mL), at 37 °C, 5% CO. The PDGCLs sphere cultures were treated in Corning^®^ Ultra-Low attachment 6 well culture plates (Sigma-Aldrich, St. Louis MO, USA). BT112 and BT179 monolayer cultures were grown in 96-well microtiter plates (Falcon; Becton Dickinson, Mylan, France) coated with laminin (Sigma-Aldrich, St. Louis MO, USA).

### Fibroblast and astrocyte cell cultures

Normal human diploid dermal fibroblasts (NHDF-c, lot #1 × 0083002.2) derived from juvenile foreskin were used as negative controls, (Promocell, Heidelberg, Germany). Fibroblast were cultured in Iscoves Modified Dulbecco’s Medium (IMDM with L-glutamine and HEPES) containing 10% fetal bovine serum, antibiotics (100 µg of penicillin and 50 µg of streptomycin sulphate/mL), and 2 mM glutamine at 37 °C, 5% CO. A second negative control was immortalized astrocytes, a gift from Dr. Linda Sleire (Department of Biomedicine, Bergen University, Norway). Astrocytes were cultured in DMEM/high glucose with L-glutamine and sodium pyruvate, non-essential amino acids 1x, 10% fetal bovine serum and antibiotics (100 µg of penicillin and 50 µg of streptomycin sulphate/mL).

### Lectin binding studies

A fluorescein-elderberry bark lectin *Sambucus nigra* (SNA) (ImmunKemi F & D AB, Järfälla, Sweden) was used as SIA expression control ligand. Glioma and fibroblast cells cultures were seeded onto 8-well glass chamber slides (Falcon, Coning, NY, USA) and the PDGCLs 2D cells were seeded onto glass chambers slices covered with laminin all of them at 37 °C for 24 h. PDGCLs spheres were cultured in Ultra-Low attachment 6 well plates. The cells were rinsed with PBS two times. Twenty µg/mL of SNA lectin in PBS was added to all monolayer cells and PDGCLs spheres which were then incubated for 2 h in the dark at room temperature. After the incubation the cells were rinsed with PBS three times. The monolayer cell cultures were fixed for 15 min in 70% ethanol and mounted in mounting medium with DAPI (Vectashield^®^, ImmunKemi F & D AB, Järfälla, Sweden). The PDGCLs spheres were fixed, frozen and then sectioned as described below. Antigen retrieval was performed by 15 min incubation in 70% ethanol. Slides were then mounted using mounting media containing DAPI. A confocal microscope (Leica SP5, with a Leica Application Suite advance florescence 2011 Software, Leica Microsystems GmbH, Wetzlar, Germany) was used for image analysis.

### GuaDex binding studies

GuaDex was prepared as described previously [9], briefly; aminoguanidine was coupled to oxidized dextran followed by reductive amination. The conjugate was purified by gel filtration on a PD-10 disposable Sephadex G-25 column (GE Healthcare, Uppsala, Sweden).

GuaDex is a cationic polydisperse carbohydrate polymer with conjugated guanidine groups. The average molecular weight is 25 kD.

Rhodamine-labelling of GuaDex was made with 500 ug rhodamine B isothiocyanate (Sigma-Aldrich, St. Louis MO, USA) mixed with 1 mL of GuaDex (15 mg/mL), in 0.02 M borate buffer at pH 9.5. FITC-labelling of GuaDex was made with 300 ug FITC (Sigma-Aldrich, St. Louis MO, USA) mixed with 1 mL of GuaDex (15 mg/mL), in 0.02 M borate buffer at pH 9.5. The solutions were incubated on a shaker, overnight in the dark at room temperature. Then the solutions were purified on a PD-10 column equilibrated with PBS. The glioma cells and astrocytes were seeded onto 8-well glass chamber slides at 37 °C for 24 h. PDGCLs spheroids were media without heparin seeded in Ultra-Low attachment 6-well plates. Rhodamine-GuaDex or FITC-GuaDex at 5 µM concentration were added to glioma cells, astrocytes, and sphere cultures. The cells were incubated 4 h in the dark at 37 °C. Then glioma cells and astrocytes were rinsed with PBS three times fixed with 70% ethanol and rinsed 2 times with PBS. Glioma cells and astrocytes were mounted on glass slides using mounting media containing DAPI. PDGCLs spheroids were fixed, frozen, sectioned and mounted on glass slides. A confocal microscope (Leica SP5) was used for the images analysis.

### Tissue fixation of PDGCLs spheres

PDGCLs spheroids after lectin and GuaDex binding were re-suspended in 4% paraformaldehyde (PFA) in PBS (pH 7.0) and incubated overnight and then transferred to a 0.5 M sucrose/PBS solution for cryoprotection. The tissue was embedded in Tissue-Tek O.C.T. compound (Sakura^®^ Finetek, Europe B.V.). Frozen sections were cut at 7 µm on a cryostat and harvested on adhesion microscope slides (Superfrost Plus™, Fisher Scientific™, Pittsburgh, PA, USA).

### Glial fibrillary acidic protein (GFAP) expression

The GFAP expression was determined to investigate if this protein could affect the GuaDex cell uptake*.* Glioma cells were seeded on glass cover slips for 24 h, formalin fixed for 10 min and washed in PBS. Cells were permeabilized with 0.1% Triton-X in PBS for 10 min at 4 °C and washed again with PBS. Frozen section antigen retrieval was performed by 20 min incubation in 10 mM citrate buffer at 90 °C after which slides were washed in PBS. Slides and cover slips were incubated with 1:500 GFAP IgG (DAKO, Z0334) in PBS, containing 0.5% BSA for 2 h at room temperature. Then the slides were washed 3 times with PBS and incubated with a secondary antibody. Texas Red goat anti-rabbit (Vectashield, Immunkemi F & D AB, Järfälla, Sweden) 1:500 was added to the sample for 1 h. Finally, the slides were washed 3 times with PBS and mounted in mounting medium containing DAPI. An inverted confocal microscope (Zeiss 710) with ZEN 2009 Software, were used for image analysis and the percentage of positive cell to GFAP expression was manually quantified.

### Fluorimetric cytotoxicity assay (FMCA)

The assay was performed as described previously [12]. Briefly, ~ 8,000 glioma or glioma patient-derived cells ~ 10,000 fibroblast or astrocytes/well were seeded onto 96-well microtiter plates (Falcon; Becton Dickinson, Meylan, France) 24 h before treatment. Temozolomide (TMZ) (Sigma-Aldrich, St. Louis MO, USA) drug control was dissolved in DMSO at 0.6 to 1.2 mM concentration. The drug control was tested as well the U343MG Cl2.6 and U1242MG and U251MG glioma cells for 24 h. Equal volume 100% DMSO and PBS were used as controls. GuaDex was tested at 5 µM incubated 1 min to 6 h and at 0.5 µM to 10 µM incubated for 24 -72 h. The control wells were furnished with PBS. All the experiments were done with six-duplicates wells. After incubation the medium was removed by flicking the plates. The cells were washed in PBS. Fluorescein diacetate (FDA) (Sigma-Aldrich, St. Louis MO, USA) was dissolved in DMSO and kept frozen at -20 °C as a stock solution (10 mg/mL). The FDA was diluted in PBS at 10 μg/mL, 200 μl was added to each well. The plates were then incubated for 30 min at 37 °C. A 96-well scanning fluorometer (Infinite^®^ M1000Pro-Tecan, Grödig/Salzburg, Austria) was used to count the emitted fluorescence of all the monolayer cells. The results were calculated using Microsoft Excel. The GuaDex CI_50_ was defined as the concentration resulting in 50% decrease in cell viability.

## GuaDex long-term effect on PDGCLs spheroids

### Fluorometric cytotoxicity assay adapted for spheroids (S-FMCA)

Spheroid viability measurements is a challenge because of the variability of spheroid morphology and varying sizes. Semi-quantitative or semi-qualitative assessment of PDGCLs viability have been used to study cytotoxicity of drugs on spheroids, measuring spheroid numbers and spheroid size, by manual counting spheroid dissociated cells after trypan blue viability staining [13], by low- or high-resolution imaging [14], and by homogeneous-sized and shaped spheroids fabrication [15]. These methods are complicated to perform and time-consuming.

A more user-friendly S-FMCA method was constructed for testing the cytotoxicity of GuaDex on spheroids and to investigate the long-term growth inhibitory effect on BT112 and BT1179 PDGCLs spheroid cultures.

The spheroid samples were incubated in 2 mL media with and without heparin (heparin being electronegative potentially could react with GuaDex) in Corning Ultra-Low attachment 6 well plates. Previous treatment, spheroids were split carefully in 2 equal volumes, one for control and the second for treatment test (2 mL each). The test spheroids were treated with 5 µM GuaDex and incubated for 48 h at 37 °C. The control wells were furnished with the media. All the experiments were done in four duplicates. After 48 h the control and test spheroids were centrifuged in tubes at 1000 rpm for 5 min, the supernatant was discarded, washed once in 1 mL PBS and then re-suspended in 2 mL new media. After 2 weeks each were split carefully into 2 equal parts. One part (1 mL) is continued 4 weeks growth and the second part (1 mL) to measure viability. The samples and controls were centrifuged (1000 rpm for 5 min) and washed in PBS as before and the supernatant discarded. A solution of 1 mL FDA (10 μg/mL) solution was added to and then gently vortexed to ensure that the spheroid cells were dissociated in suspension for incubation at 37 °C for 30 min. Then the tubes were gently vortexed again to ensure that all the spheroid cells were in a suspension and dispersed in a cuvette. The S-FMCA was measured at 490 nm using a spectrophotometer (Genesys™ 10S Thermo Scientific™, Waltham, MA, USA). The remaining spheroids were re-plated to grow in 2 mL new media for a total of 4 weeks. Then, each full volume sample (2 mL) was collected and repeated the procedure as after 2 weeks incubation preparing each sample for S-FMCA measurement.

Images of the PDGCLs spheroids incubated with GuaDex were obtained following the experiment of the S-FMCA measurement. An inverted microscope Leica DM IL LED, (Leica microsystems, Wetzlar, Germany) was used to obtain images at 10 min, 24 h, 1- 2- and 4-weeks incubation of GuaDex on BT112 and BT179 PDGCLs spheroids.

## Results

### Monolayer cells

**Fig. 1:**
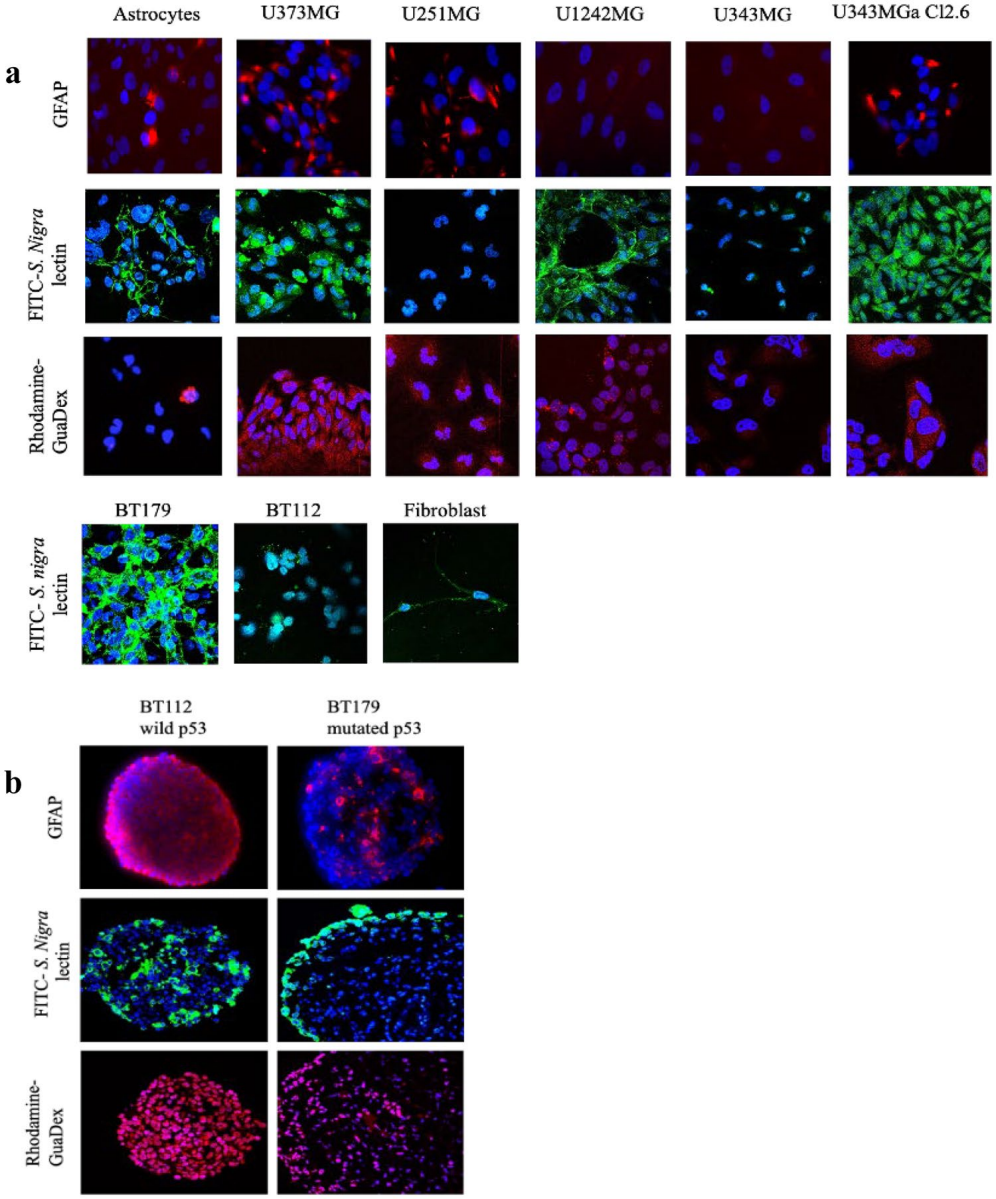
Confocal microscopy images: **a**) monolayers glioma cell lines, GFAP expression, Sialic acid α-2,6-Gal expression FITC-labelled *S. nigra* lectin, Rhodamine-GuaDex binding, monolayer BT112 and BT179 PDGCLs, and fibroblasts, sialic acid expression, DAPI staining, nucleus (blue); **b**) corresponding at BT112 and BT179 PDGCLs spheroids. Magnification 40x

In general, the cellular uptake of GuaDex was enhanced and correlated with the expression of Sia but also to some extent with GFAP expression (fluorescent labelled GuaDex, Fig. 1). Astrocytes and U343MG with low/no expression of these markers showed very low uptake of GuaDex (Fig. 1a).

### Spheroids

Similar correlation was observed in spheroids. BT179 with low expression of both markers showed low uptake of GuaDex. Larger spheroids (~ 0.8 mm) showed less uptake compared to smaller spheroids (~ 0.5 mm) showing strong uptake (Fig. 1b).

### GuaDex-FITC tumor cell interaction

**Fig. 2:**
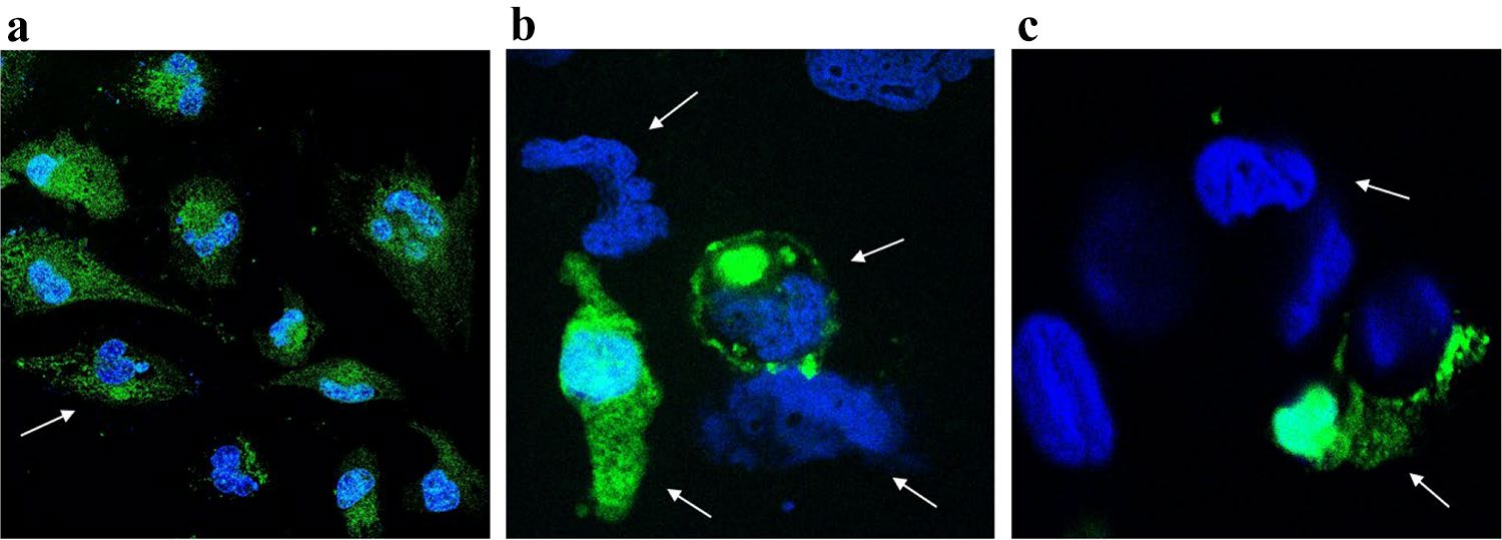
Confocal microscopy, images at 4 h incubation at 5 µM FITC-GuaDex U343MG binding, **a**) membrane adsorption; **b**) and **c**) absorption /internalization, nuclear membrane perforations, nuclear fragmentation, cell membrane collapse and nuclear collapse/DNA precipitation. DAPI staining, nucleus (blue). Magnification 40x

The Fig. 2 showed the mechanism of the interaction of GuaDex-FITC with U343MG cell membrane and nuclear membrane.

### Growth inhibition efficacy

**Fig. 3:**
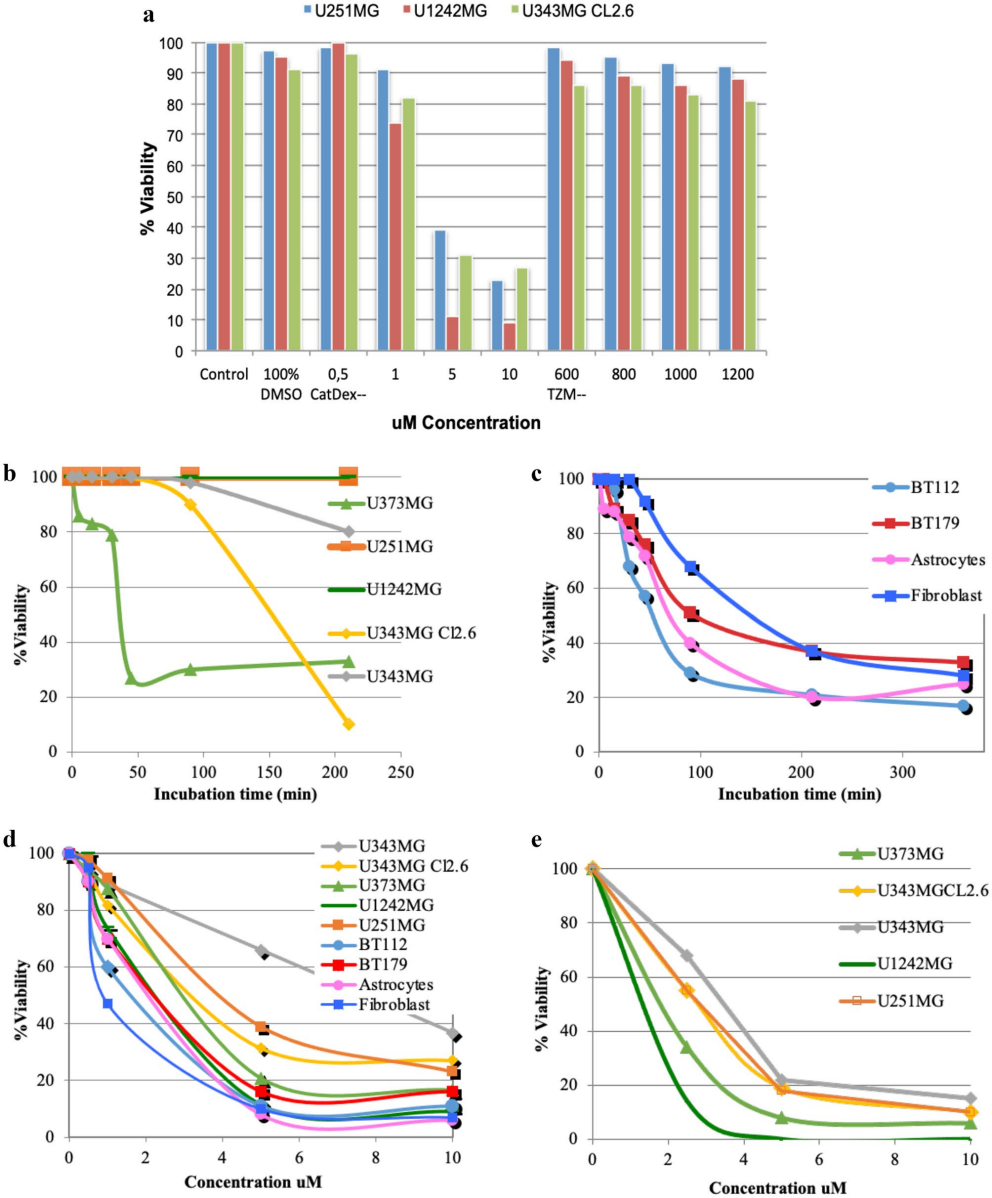
FMCA cytotoxicity assay, **a**) GuaDex and temozolomide compared, 24 h incubation at different concentrations. Control, 100% DMSO; **b**), **d**) and **e**) monolayer glioma cells incubated at 5 µM GuaDex during 3.5 h, 24 h, and 72 h.; **c**) and **d**) monolayer BT112 and BT179 PDGCLs, astrocytes and fibroblast incubated at 5 µM GuaDex during 6 h and 24 h

GuaDex showed superior efficacy compared to TMZ where TMZ showed no effect at equimolar concentrations (0.5–10 µM). TMZ required 100 times higher concentration to show efficacy (Fig. 3a). GuaDex efficacy was time and concentration dependent (Fig. 3b, c, d and e). The GuaDex IC_50_ at treatment on glioma cells lines was 2.2- 7.4 µM at 24 h and 1.2–3.0 µM at 72 h. The IC_50_ at 24 h was 2.2–2.5 µM on monolayer glioma patient-derived cell lines (PDGCLs).

### Lasting growth inhibition on PDGCLs spheroids

**Fig. 4:**
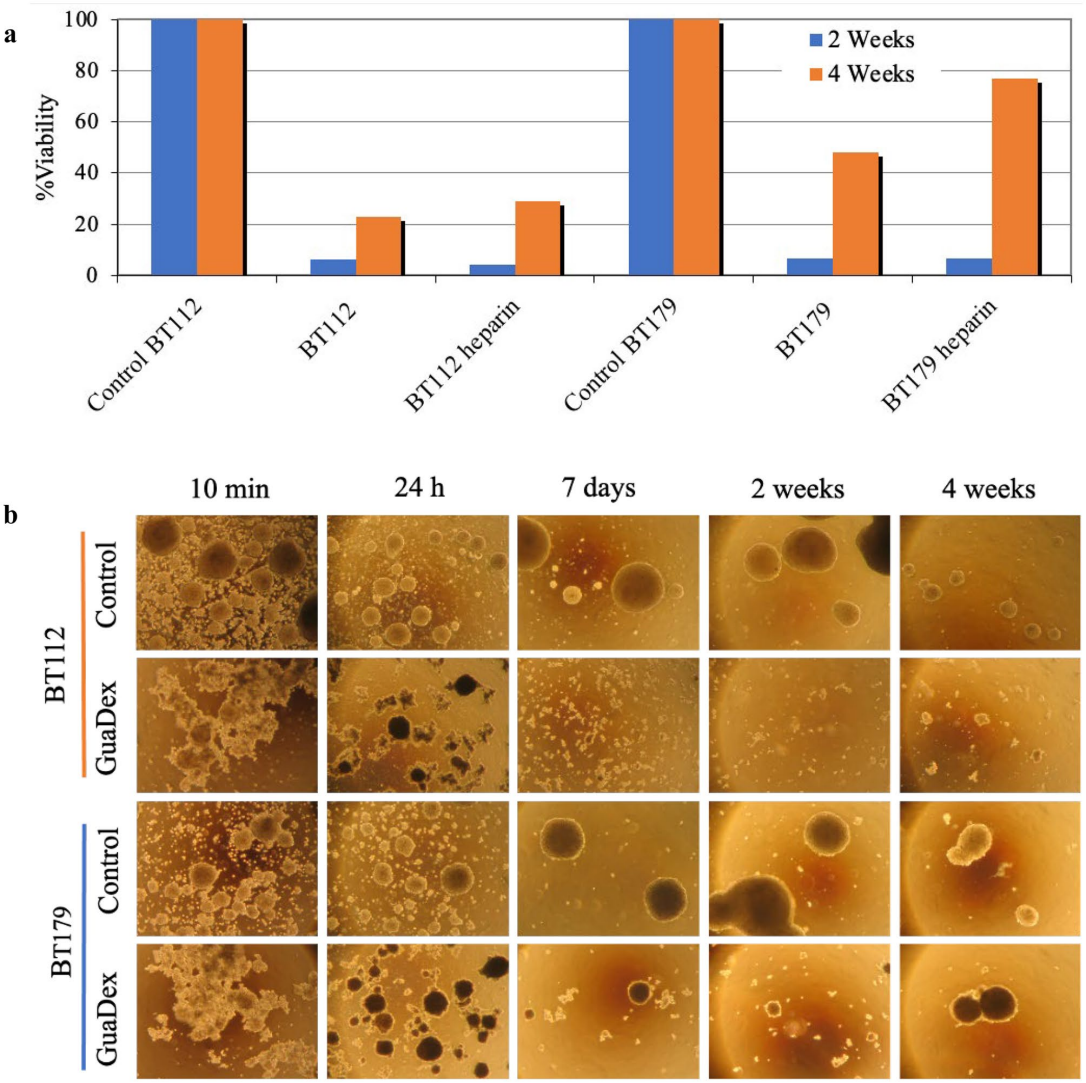
**a**) Spheroid fluorometric cytotoxicity assay (S-FMCA), 2 and 4 weeks, long-term growth inhibitory effect after an initial 48 h incubation at 5 µM GuaDex concentration, with and without heparin containing media, BT112 and BT179 PDGCLs spheroids. **b**) Inverted microscope images, long-term inhibitory effect after an initial 48 h incubation at 5 µM GuaDex concentration, without heparin containing media on BT112 and BT179 PDGCLs spheroids. Magnification 20x

GuaDex was incubated with the spheroids for 48 h, washed and then tested for viable cells after 2 weeks and 4 weeks post treatment. The results showed a prolonged growth inhibition effect, < 7% viable, 2 weeks post treatment and 23–48% viable cells after 4 weeks post treatment. If media contained heparin, there seemed to be certain inhibition of the GuaDex efficacy (heparin is electronegative may interact with GuaDex). There was no regrowth in BT112 while BT179 showed new proliferation (Fig. 4a and b).

### GuaDex

**Fig. 5:**
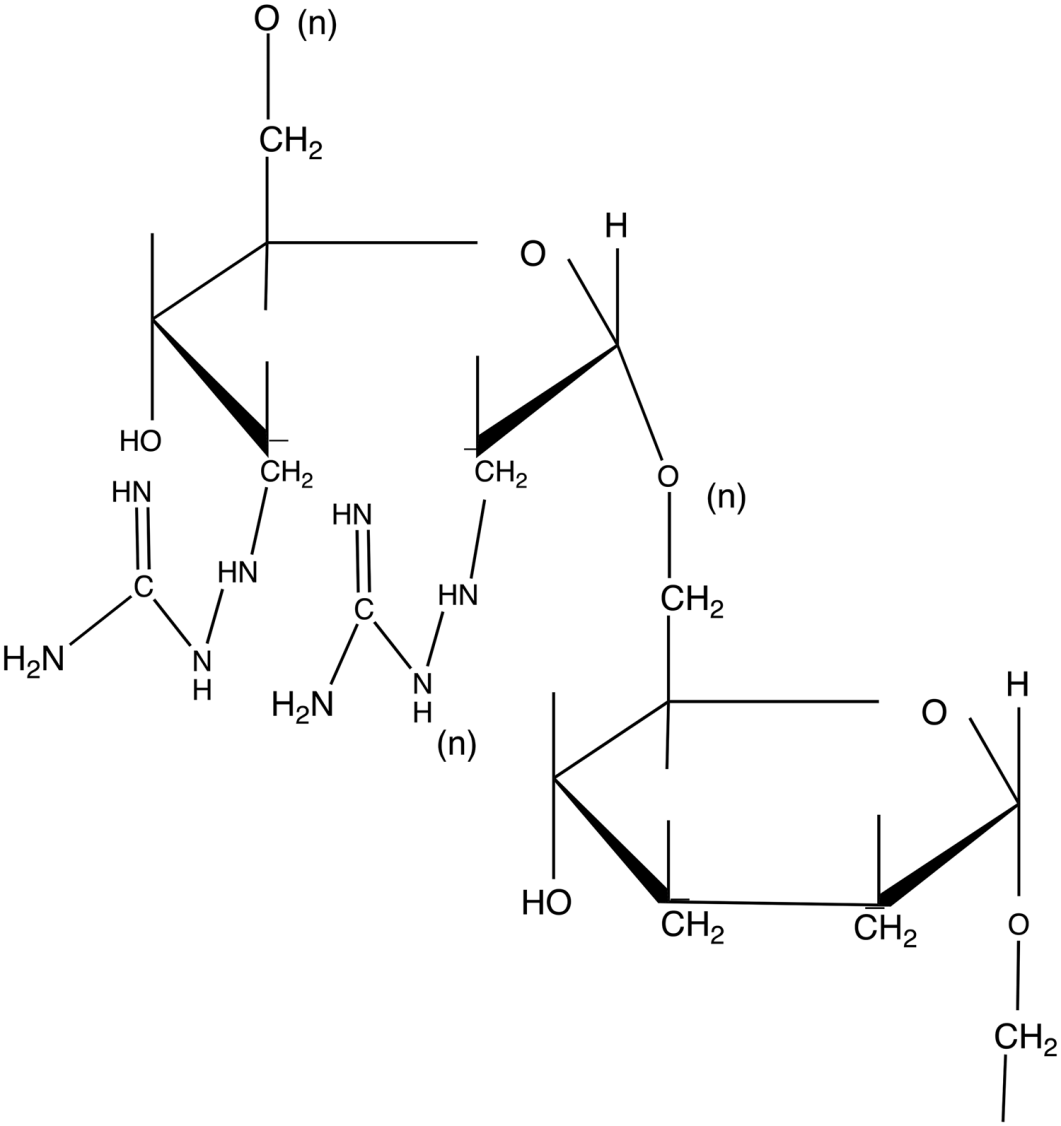
Schematic molecular formula of GuaDex

The schematic molecular formula of GuaDex is illustrated in Fig. 5.

## Discussion

During the last decade there has been an upsurge in research on GBM resulting in new insights in molecular mechanisms and genetic mutations/changes. New therapeutic strategies have been suggested and are actively being investigated, such as gene therapy, immunotherapy, and stem cell therapy [16]. There are also several targeted therapies that have been put forward for consideration as novel treatment options, for example immune checkpoint targeting inhibitors [17]. The use of nano technology for the delivery of cytotoxic entities to the GBM target has also been suggested [18]. The use of electric fields applied by an external device, tumor treating fields (TTF), has been developed with some positive results [19].

However, despite considerable improvement of the understanding of the GBM pathology with several connected treatment suggestions and further developments in radiosurgery & radiation technology, GBM resists treatment and prognosis remains poor.

There are obviously a number of circumstances and reasons for this apparent lack of significant progress in GBM treatment. Maybe the most important obstacle is its delicate location together with its infiltrative growth pattern, surrounded with vital brain tissue precluding radical interventions. Further complicating is the BBB that limits the possibilities for systemic therapy although there are claims that BBB is disrupted in GBM allowing systemic therapy [20].

However, there are also several other limiting factors that explain the shortage of new effective GBM therapies. Translational cancer drug research usually means the translation from "the lab bench" to clinical research that eventually may result in a market approved new prescription drug. This translation is a most difficult endeavour with low, or very low success rates. The translation process of novel cancer drug candidates is described as "crossing the valley of death" i.e., death of drug candidates [21]. The US food and drug administration (FDA) and European medicines agency (EMA) usually require animal studies before a cancer drug candidate may enter human clinical research but less than 8% translates in human positive results in clinical trials. Of those projects that enter clinical research 85% fail in the early phases and of those that make it to phase III only 50% are finally approved for clinical use [22]. The same very modest success rates are noted also for immunotherapies (e.g., cancer vaccines, immune checkpoint inhibitors). As an example, of 23 phase II/III clinical trials testing cancer vaccines, 78% failed [23]. Besides these rather discouraging figures, the financial costs for translational research are huge. The cost for successful translational development resulting in an approved novel drug exceeds US $ 1 billion [24, 25].

In view of these facts and circumstances the shortage of novel drugs for GBM might not be surprising.

The results from this in vitro study show that GuaDex, a cationic polymer with guanidine side-groups, has a potent cytotoxic effect on glioma cells including glioma cell spheroids. The mode of action appears very similar to that of tumor treating fields (TTS) affecting the intrinsic electrostatic properties of the tumor cells and like this disrupting their integrity [26].

The effect was observed at low µM concentrations (IC50 <  ~ 3 µM) in contrast to the positive control (TMZ) that only showed a slight cytotoxic effect even at mM concentration. TMZ was dissolved in DMSO (dimethyl sulfoxide), an amphiphilic solvent. DMSO is frequently used in cell biology as enhancer of cell membrane penetration [27]. DMSO had by itself a slight cytotoxic effect (Fig. 3) and the difference to TMZ dissolved in DMSO was small.

In view of GuaDex significant in vitro GBM toxicity, the question is how it might be applied clinically for GBM therapy. GuaDex might not seem suitable for systemic administration because of BBB restriction owing to its chemical properties (size, charge etc.). Local application as an interstitial therapy might be feasible in connection with initial surgery of unifocal GBM. Local delivery of chemotherapeutic agents has been explored previously. Biodegradable wafers (Gliadel) with adsorbed carmustine implanted in connection with surgery have been studied in several human trials. Carmustine is a nitrosourea DNA alkylator. The wafers release the carmustine during a period of several weeks, i.e. a slow-release formulation. There appears to be some survival benefit for certain patient groups receiving this interstitial therapy [28]. The most common side effects with Gliadel implants include brain edema with convulsions, hemiparesis, aphasia, infection, and visual field defects [29]. Since in most cases, GBM recurrence seems to occur in the wall of the resection cavity within 20 mm of its margin, interstitial therapy appears to be attractive and an opportunity to prevent recurrence [30]. GuaDex could be adsorbed into a biodegradable/biocompatible matrix, implanted after surgery, and then it would gradually diffuse into the surrounding interstitial space. Although the concept of interstitial chemotherapy appears attractive and strait forward, there are several circumstances that may hamper successful targeting of remaining infiltrating tumor cells post-surgery. Increased interstitial pressure due to edema resulting from the tumor itself, as result of the surgery and maybe aggravated by the implant, can make efficient diffusion difficult to achieve. Furthermore, the anatomy of the brain interstitial space is tortuous, long and complex with hindrances to diffusion e.g., local limits ("dead ends") and local viscosity [31, 32] and, changes because of the GBM pathology. In spite of these examples of unfavourable factors that may hinder diffusion, GuaDex chemical properties might be an advantage, namely its strong cationic electrostatic charge, anti-microbial properties [33] and its molecular polydispersity. When GuaDex is in the interstitial space, there appears to be good possibilities for charge specific targeting of the electronegative GBM cells with subsequent tumor cell toxicity. This study indicates that the glioma electronegativity i.e., cell surface hypersialylation, contribute to the efficacy of GuaDex. Tumor cells with high sialyl expression and/or positive intracellular GFAP expression yield faster induction of GuaDex toxicity. Glial fibrillary acidic protein, GFAP, is thought to help to maintain astrocyte mechanical strength as well as the shape of cells, but its exact function remains poorly understood [34].

The feasibility of targeting electronegative tumor cells with a cationic polymer has been confirmed before [8]. Polymers with different charge were delivered into the urinary bladder through instillation on patients with superficial bladder cancer. The result showed very high tumor accumulation of cationic polymers and low uptake in normal tissue [8].

GuaDex polydispersity i.e., consisting of a cocktail of multiple molecular species, ranging from low molecular weight (mw) to higher mw, might facilitate diffusion/penetration into the heterogeneous and complex interstitial space. In addition, its apparent anti-microbial properties might decrease the risk of causing infection during the instillation [33].

In summary. In order to translate and test GuaDex in vitro efficacy into clinical studies, briefly, further pre-clinical studies need to be done, in particular determination of the safety i.e., possible toxicity of intra CNS instillation and how optimal diffusion into the surrounding interstitial space should be achieved. Development of a suitable biocompatible/degradable absorbent needs to be accomplished. Fast advancement to Phase 0 trials [35] would be desirable due to the limited relevance of pre-clinical in vivo models.

## Declarations

### Authors' information

Not special remarks.
